# Stratosphere Conditions Inactivate Bacterial Endospores from a Mars Spacecraft Assembly Facility

**DOI:** 10.1089/ast.2016.1549

**Published:** 2017-04-01

**Authors:** Christina L. Khodadad, Gregory M. Wong, Leandro M. James, Prital J. Thakrar, Michael A. Lane, John A. Catechis, David J. Smith

**Affiliations:** ^1^ Sierra Lobo, Inc., Kennedy Space Center, Florida.; ^2^ Department of Geosciences, Pennsylvania State University, University Park, Pennsylvania.; ^3^ NASA, Engineering Directorate, Kennedy Space Center, Florida.; ^4^ NASA, Space Biosciences Division, Ames Research Center, Moffett Field, California.

## Abstract

Every spacecraft sent to Mars is allowed to land viable microbial bioburden, including hardy endospore-forming bacteria resistant to environmental extremes. Earth's stratosphere is severely cold, dry, irradiated, and oligotrophic; it can be used as a stand-in location for predicting how stowaway microbes might respond to the martian surface. We launched E-MIST, a high-altitude NASA balloon payload on 10 October 2015 carrying known quantities of viable *Bacillus pumilus* SAFR-032 (4.07 × 10^7^ spores per sample), a radiation-tolerant strain collected from a spacecraft assembly facility. The payload spent 8 h at ∼31 km above sea level, exposing bacterial spores to the stratosphere. We found that within 120 and 240 min, spore viability was significantly reduced by 2 and 4 orders of magnitude, respectively. By 480 min, <0.001% of spores carried to the stratosphere remained viable. Our balloon flight results predict that most terrestrial bacteria would be inactivated within the first sol on Mars if contaminated spacecraft surfaces receive direct sunlight. Unfortunately, an instrument malfunction prevented the acquisition of UV light measurements during our balloon mission. To make up for the absence of radiometer data, we calculated a stratosphere UV model and conducted ground tests with a 271.1 nm UVC light source (0.5 W/m^2^), observing a similarly rapid inactivation rate when using a lower number of contaminants (640 spores per sample). The starting concentration of spores and microconfiguration on hardware surfaces appeared to influence survivability outcomes in both experiments. With the relatively few spores that survived the stratosphere, we performed a resequencing analysis and identified three single nucleotide polymorphisms compared to unexposed controls. It is therefore plausible that bacteria enduring radiation-rich environments (*e.g.,* Earth's upper atmosphere, interplanetary space, or the surface of Mars) may be pushed in evolutionarily consequential directions. Key Words: Planetary protection—Stratosphere—Balloon—Mars analog environment—E-MIST payload—*Bacillus pumilus* SAFR-032. Astrobiology 17, 337–350.

## 1. Introduction

Preventing the forward contamination of Mars is required for United States and international space missions (NASA, 2005; Kminek and Rummel, 2015). Yet spacecraft leaving Earth still carry microorganisms on board that are embedded within surfaces, instruments, electronics, and other inaccessible areas that cannot be readily cleaned (Schuerger *et al.,* 2003; Nicholson *et al.,* 2005; Benardini *et al.,* 2014). Complete spacecraft sterilization has not been enforced since the Viking missions. Currently allowable microbial bioburden on spacecraft, while relatively low (Benardini *et al.,* 2014), makes the pristine martian environment vulnerable to contamination. Moreover, future life-detection missions could be threatened by false positives without a better understanding of which microorganisms are most capable of persistence, growth, or replication once delivered to the Red Planet. A recent analysis from the Second MEPAG Special Regions Science Analysis Group (Rummel *et al.,* 2014) identified major knowledge gaps associated with polyextremophiles (microorganisms resistant to more than one environmental stressor), particularly when shielded from UV light on Mars by global dust storms, regolith, or overlying dead microorganisms. Nicholson *et al.* (2005) and Horneck *et al.* (2010) reviewed common bacterial adaptations to extreme environments, including sporulation, cell pigmentation, and DNA repair pathways (*e.g.,* homologous recombination and nonhomologous end joining). Nonsporulating bacteria also have various mechanisms with which to resist damaging radiation in the space environment. For example, *Deinococcus radiodurans* can repair its DNA through homologous recombination (Krisko and Radman, 2013). Other microbes, such as a halophilic *Synechococcus* species, can shield biomolecules from radiation with exogenous salt crystals (Mancinelli *et al.,* 1998).

Smith (2013) argued that Earth's stratosphere would allow multiple Mars-like conditions to be simultaneously tested if polyextremophilic species could be exposed to the upper atmosphere and returned for analysis. Recent missions have demonstrated the feasibility of transporting biological samples to the stratosphere by using small, meteorological balloons (Beck-Winchatz and Bramble, 2014) and large scientific balloons (Smith *et al.,* 2014). The pressure of the thin and dry stratospheric air around 25–38 km above sea level (ASL) is roughly equivalent to the surface pressure on Mars (0.5–1 kPa). The stratosphere is also a cold and extremely dry environment with elevated levels of ionizing and non-ionizing radiation (Adams *et al.,* 2007; Dachev, 2013; Schuerger and Nicholson, 2016). Relative humidity levels can drop below 1%, and temperatures in the lower stratosphere regularly reach −100°C. Stratospheric radiation is substantially higher (Kylling *et al.,* 2003; Adams *et al.,* 2007; Dachev, 2013) than doses at other frequently visited Mars analog environments, including the McMurdo Dry Valleys of Antarctica (Wynn-Williams and Edwards, 2000) and the Atacama Desert (McKay *et al.,* 2003). Laboratory environmental chamber experiments have been employed in the past to simulate martian conditions (Schuerger *et al.,* 2003; Diaz and Schulze-Makuch, 2006; Tauscher *et al.,* 2006; de la Vega *et al.,* 2007; Moores *et al.,* 2007; Osman *et al.,* 2008; Fendrihan *et al.,* 2009; Smith *et al.,* 2009; Gómez *et al.,* 2010; Peeters *et al.,* 2010; Johnson *et al.,* 2011; Kerney and Schuerger, 2011), but artificial illumination sources do not realistically represent the dynamic nature of sunlight. Moreover, most ground-based simulation studies do not simultaneously create the full range of biological stressors present on Mars (*i.e.,* hypobaria, desiccation, irradiation, nutrient deprivation, oxidation, and low temperatures). Conveniently, Earth's upper atmosphere produces a natural combination of these extreme conditions. Measuring the response and survival of polyextremophilic species in the stratosphere can therefore be used to test Mars forward contamination scenarios.

On 10 October 2015, we flew a balloon experiment to the stratosphere over New Mexico and Texas (United States) that reached an altitude of 31.4 km ASL. The Exposing Microorganisms in the Stratosphere (E-MIST) payload carried known quantities of bacterial endospores (hereafter referred to as “spores”) to the Mars-like environment for 2, 4, 6, and 8 h exposures. We used a spacecraft assembly facility–isolated bacterial strain *Bacillus pumilus* SAFR-032 for the balloon flight (and subsequent ground experiments). The Gram-positive, aerobic, endospore-forming bacterium is noteworthy for special resistance to desiccating, UV-intense conditions (Link *et al.,* 2004; Kempf *et al.,* 2005; Gioia *et al.,* 2007; Tirumalai *et al.,* 2013). Since the spores used for the balloon experiment were metabolically dormant, exposure to stratospheric conditions resulted in cumulative damage to cellular components that was measurable in the laboratory after the E-MIST payload returned to the ground. Our experimental design was inspired by similar experiments with *B. pumilus* SAFR-032 spores outside the International Space Station (ISS) (Horneck *et al.,* 2012; Moeller *et al.,* 2012; Nicholson *et al.,* 2012; Vaishampayan *et al.,* 2012).

Sending known quantities of viable, monolayered spores into Earth's stratosphere and making comparisons with unexposed controls allowed us to (1) determine the survival of spore populations using culture-based enumeration methods and (2) assess genomic alterations through a resequencing analysis of surviving spores. We also collected environmental data during the balloon flight and performed supplemental ground UV experiments, with an overall goal of assessing the survivability of *B. pumilus* SAFR-032 spores in stratospheric conditions that closely resemble the surface of Mars.

## 2. Materials and Methods

### 2.1. Bacterial strain description and sample preparation

*Bacillus pumilus* SAFR-032 spores were also used for the first E-MIST balloon test flight (Smith *et al.,* 2014). A full genome map was available (NCBI, GCA000017885.4 ASM1788v4) with a total of 3819 genes previously identified for the species (Gioia *et al.,* 2007). The strain was safe to work with in the field (Biosafety level 1; no hazard posed to balloon personnel or the environment), and prepared spores were stable in stasis, allowing for simplified mission logistics. Moreover, there was no exosporium or extraneous biofilms/layers associated with spores resulting in straightforward postflight molecular assays (Link *et al.,* 2004; Gioia *et al.,* 2007; Vaishampayan *et al.,* 2012; Tirumalai *et al.,* 2013). Testing SAFR-032 spores also enabled comparisons with past experiments that used the model microorganism (Horneck et *al.,* 2012; Moeller *et al.,* 2012; Nicholson *et al.,* 2012; Vaishampayan *et al.,* 2012; Smith *et al.,* 2014).

We established a spore stock based on previously established methods (Schaeffer *et al.,* 1965; Nicholson and Setlow, 1990; Vaishampayan *et al.,* 2012) by culturing the original isolate of *B. pumilus* SAFR-032 in Difco nutrient broth and incubating at 35°C, 140 rpm for 16 h. Germinated cells were then transferred to sterile sporulation media with the following nutrients per 1 L sterile nanopure water: 8 g Difco nutrient broth, 1 g potassium chloride, and 0.25 g magnesium sulfate heptahydrate autoclaved together followed by the addition of 1 mL sterile calcium chloride (7.35 g/100 mL), manganese chloride (0.2 g/100 mL), and ferrous sulfate (0.0278 g/100 mL). The culture was incubated for 124 h at 35°C with shaking at 140 rpm. Resultant spores were then divided evenly into 50 mL tubes (containing ∼40 mL of culture each) and submerged into a water bath at 80°C for 15 min to destroy any remaining vegetative cells, followed by centrifugation for 20 min at 9400 RCF. Next, heat-treated spores were washed by resuspension with sterile molecular-grade water, centrifugation, and removal of supernatant. This process was repeated four times. After the final wash, spores were resuspended in 8 mL of sterile molecular-grade water and stored overnight in a cold incubator at 4°C with shaking at 90 rpm. To remove any cellular debris, we repeated the cold incubation and wash cycle three consecutive times. Once completed, the supernatant was removed, and the pellet containing purified spores was transferred to a sterile glass test tube containing 0.5 mL sterile molecular-grade water.

**FIG. 1.**
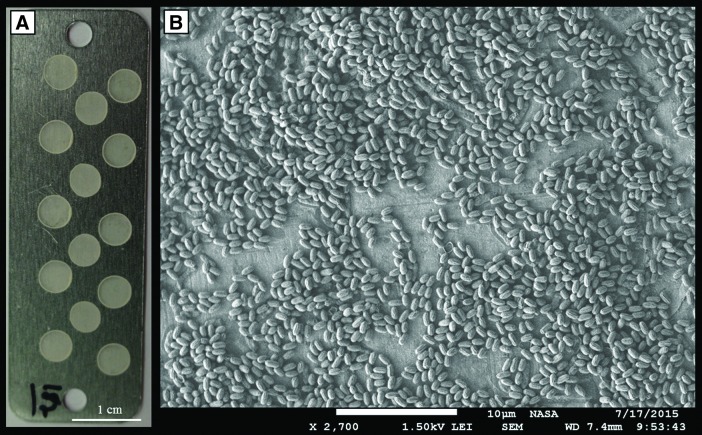
(**A**) An experimental coupon with 14 separate *B. pumilus* SAFR-032 spore aliquots; each aliquot contained approximately 4.07 × 10^7^ spores total. (**B**) Scanning electron micrograph of dried *B. pumilus* SAFR-032 spores distributed within a single 20 μL coupon aliquot.

Spore stock concentration was determined to be approximately 6.29 × 10^9^ spores per 1 mL through the most probable number (MPN) method and heterotrophic plate counts. Dilutions from the original stock (20 μL aliquots) were then seeded onto sterile aluminum coupons (M4985, Seton) and dried for 4 h in a dark laminar flow hood at 25°C, creating a layer of 4.07 × 10^7^ spores adhering to coupon surfaces for each individual aliquot. A total of 14 separate 20 μL aliquots were deposited onto any single experimental coupon (5.40 × 1.75 × 0.51 cm) (Fig. 1A). Experimental coupons were created in the same batch, then stored in sterile, dark containers. Dried coupons were imaged with a scanning electron microscope (JSM-7500F, JEOL) to assess the distribution of spores on the aluminum coupon surface (Fig. 1B). Recovery of spores from experimental coupons was achieved through polyvinyl alcohol (PVA) film peels (Horneck *et al.,* 2001; Moeller *et al.,* 2012). Twenty microliters of sterile 10% PVA prepared in water was applied in a thin layer over the dried spores previously deposited onto each coupon. After drying in an incubator for 1 h at 37°C, the PVA film contained embedded spores and was peeled off the coupon with sterile forceps. The PVA film was dropped into a glass test tube containing sterile molecular-grade water and resuspended with a vortexer.

### 2.2. Enumeration and resequencing assays

In general, microbial coupons from flight experiments and ground simulations were assayed to (1) determine the number of viable surviving spores (compared to starting quantities) and (2) assess the extent of nonlethal genetic mutations through resequencing (compared to controls and the reference *B. pumilus* SAFR-032 genome). The MPN enumeration method originally described by Mancinelli and Klovstad (2000) and modified by Schuerger *et al.* (2003) was used for this study. After incubation at 30°C for 36–42 h, plate wells were scored for growth and calculated to MPN values. To analyze the genome of surviving spores, we followed a protocol from the work of Nicholson *et al.* (2012). After the PVA peel step, spore suspensions were distributed into 5 mL of recovery media (1:9 ratio of 10 m*M* L-alanine:2X LB) and then incubated at 37°C with shaking at 250 rpm for 60 min allowing approximately one cell replication cycle. Next, germinated cultures were centrifuged at 5000 rpm for 10 min. The supernatant was removed by pipette, and pellets were resuspended in 1.8 mL of sterile molecular-grade water for DNA extraction with the UltraClean Microbial DNA Isolation Kit (MoBio). Tubes were centrifuged at 14,000 rpm for 3 min with the exception of the microbead and filter tubes, which were spun at 10,500 rpm. Rather than vortexing the microbead tubes for 10 min, the tubes were placed in a Biospec benchtop minibead beater for 1 min. The extracted DNA was then quantified with a Qubit 2.0 Fluorometer and a high-sensitivity DNA assay (Invitrogen). All DNA samples were stored at −20°C. Purities of the spore stock and batch-produced experimental coupons were monitored throughout the study with heterotrophic plate counts and DNA sequencing—no contaminating species were identified.

Sequencing was conducted by using the Illumina Nexterra XT kit (Illumina). First, 1 ng of DNA was tagged with indices followed by a 5 min temperature cycling of 55°C and 10°C to fragment the DNA. Amplification followed, cycling at 72°C for 3 min, 95°C for 30 s, and 12 cycles of 95°C for 10 s, 55°C for 30 s, and 72°C for 30 s. A final elongation step at 72°C for 5 min completed the cycle. Amplified DNA was cleaned with the MinElute PCR cleanup kit (Qiagen) and denatured with 0.2 *N* NaOH and heating per Illumina Protocol #15044223 Rev B. Sequencing reads were run on the Illumina MiSeq and prepared with the Illumina V2 300 kit. To identify and assemble contigs, data were analyzed with CLC Workbench v 8.0.2 (Qiagen Bioinformatics). The software located single nucleotide polymorphisms in samples compared to the *B. pumilus* SAFR-032 reference genome. Nucleotide variant type (*i.e.,* deletion, insertion, or substitution) and the frequency across samples were mapped to known coding regions for the strain.

### 2.3. E-MIST hardware description

Several noteworthy hardware modifications (Supplementary Figs. S1 and S2; Supplementary Figures are available at www.liebertonline.com/ast) were made after the first E-MIST test flight described by Smith *et al.* (2014). Four independently rotating skewers fitted with an adjustable aluminum sample base plate allowed an exposure time series to be established in the stratosphere. Each sample plate held 10 separate aluminum coupons (see Section 2.1). The plates were enclosed within Nomex-lined cylinders to prevent sunlight from entering during the balloon gondola ascent/descent and when the skewers rotated to a closed position for the experimental time series. Each skewer was motor-controlled (SPG30E-300K, Cytron) by a 4-channel motor driver (FD04A, Cytron) held together by an aluminum and polycarbonate frame. Multiple instruments were inside the payload housing, including a GPS unit (SPK-GPS-GS4O7A, S.P.K. Electronics Co.), a radiometer with UV sensors (PMA2100, PMA2107, and PMA2180, Solar Light), and an external humidity and temperature sensor (HOBO, U23-001, Onset). Instrument temperatures were regulated inside the payload with heating pads (5V, WireKinetics). The avionics system (chipKIT Max32, Digilent) used a serial peripheral interface connection to communicate with a micro-SD card (BOB-00544, microSD Transflash Breakout, SparkFun) and a micro DB-9 port (1200-1183-MIL, Digi-Key). A 1080P HackHD camera was controlled by the avionics and recorded imagery throughout the flight. Other major hardware components included an altimeter (MS5607, Parallax), 8.5 W heaters (Omegalux Kapton Insulated Flexible Heater, Omega), and multiple resistance temperature detectors (SA1-RTD-B, Omega). Power was generated by a 14.8v 25.2 Ah lithium-ion polymer battery (CU-J141, BatterySpace). Before flying, the E-MIST payload was subjected to vibration and hypobaric chamber testing to validate system performance. Instruments were recalibrated after testing, and then the payload was shipped to the launch site at Ft. Sumner, New Mexico, where it was mounted onto the uppermost gondola portion of Columbia Scientific Balloon Facility (CSBF) long-duration balloon Test Flight II (#667NT). Prelaunch, landing, and recovery procedures described by Smith *et al.* (2014) were repeated for this experiment. Prior to installing base plates onto the skewers with *B. pumilus* SAFR-032 coupons, the inside of skewer canisters were sprayed with sterile air, and payload surfaces were wiped with isopropyl alcohol.

### 2.4. Experimental design

Ten experimental coupons were attached (in random order) to four separate base plates on the rotatable E-MIST skewers. Each coupon contained 14 identically prepared 20 μL aliquots of *B. pumilus* SAFR-032 deposited at a concentration of 4.07 × 10^7^ spores per aliquot. This arrangement allowed for a high number of potential replicates per skewer (*N* = 140). All skewers were opened simultaneously in the stratosphere, then closed sequentially: Skewer 1 exposed samples for 2 h; Skewer 2 exposed samples for 4 h; Skewer 3 exposed samples for 6 h; and Skewer 4 exposed samples for 8 h. Two different sample coupon orientations were established on each base plate to determine the effect of stratospheric conditions with and without sunlight, as follows: (1) nine coupons were mounted upright and exposed directly to sunlight, and (2) one coupon was mounted upside-down (inverted) to prevent illumination. A blank negative control coupon was also located on the payload and used for monitoring potential contamination associated with launch and landing. To measure the possible influence of transportation to the field site and/or delays associated with balloon flight operations, we included two sets of ground controls—coupons that traveled to Ft. Sumner, New Mexico, but were not flown, and another set that remained in the laboratory at Kennedy Space Center (KSC), Florida.

### 2.5. Ground experiments

To supplement the balloon flight experiment, we used another group of coupons to evaluate *B. pumilus* SAFR-032 spore resistance to artificially generated UVC conditions in the laboratory. Within a biological safety cabinet (Labgard Class II, Type A/B3, Model NU-600 Series 24, NuAire, Inc.), a 3-D-printed acrylic UV light-emitting diode (LED) test stand (12.0 × 7.5 × 14.1 cm) held experimental coupons at vertical distances of 1–5 cm from the light source (Supplementary Fig. S3). The test stand bridge could vertically move the LED at half-centimeter intervals and change the angle of illumination 45–90° relative to the sample base plate below. A UVC LED (Part # UVTOP270TO39FW, QPhotonics, LLC) with 5.668 V and a maximum current setting at 20.00 mA generated a peak wavelength of 271.1 nm and a spectral width of 10.3 nm. A Light Meter (HHUV254SD, Omega) was used to measure maximum UVC intensities of 0.50, 0.17, 0.090, 0.040, and 0.030 W/m^2^ for coupons located 1, 2, 3, 4, and 5 cm from the LED, respectively. The first ground experiment exposed bacterial coupons (4.07 × 10^7^ spores per aliquot) to UVC at distances of 1–1.5 cm (resulting in 0.50–0.27 W/m^2^) for durations of 1.3, 6.7, 50, 100, and 240 min. For each run, the angle of incidence for coupon illumination was 90° from the plane of the UVC LED. Samples were enumerated after the UVC exposure by using the MPN procedure described in Section 2.2. A second ground experiment was also conducted with a lower starting concentration of *B. pumilus* SAFR-032 spores (640 spores per aliquot). Samples were exposed for 15 min at 90° from the UVC LED plane in distances of 1–5 cm (0.50–0.030 W/m^2^). Next, samples at a distance of 5 cm (0.030 W/m^2^) were exposed for 0–25 min at 90° from the UVC LED. The final portion of the experiment exposed samples for 15 min at a distance of 5 cm from the UVC LED, but the orientation of the light source was changed to create 45°, 60°, 75°, and 90° angles of incidence relative to the plane of the coupon.

### 2.6. Statistical analyses

Means and standard errors were calculated for samples from each flight experiment treatment (2, 4, 6, and 8 h). For every group, we sampled three random coupons, with three separate bacterial aliquots processed from each coupon; this provided a total of *N* = 9 replicates per group. Fewer inverted test coupons were flown (only one coupon per skewer), so samples were enumerated in triplicate. Our UVC ground experiment had five time treatments with *N* = 9 replicates from each group. The second UVC experiment (with a lower starting concentration of spores) processed samples in triplicate and had independent treatments for time (0–25 min), distance (1–5 cm), and angles of incidence (45–90°). To analyze values from both flight and ground experiments, we ran one-way ANOVA analyses, producing *P* values at 95% confidence levels to determine whether viability numbers were changing significantly compared to initial quantities. *F* values were also calculated to estimate the variance of MPN values between and within groups. Finally, a Tukey HSD test compared individual sample group means by using the same confidence level applied to *F* and *P* tests.

## 3. Results

### 3.1. Description of balloon flight

**FIG. 2.**
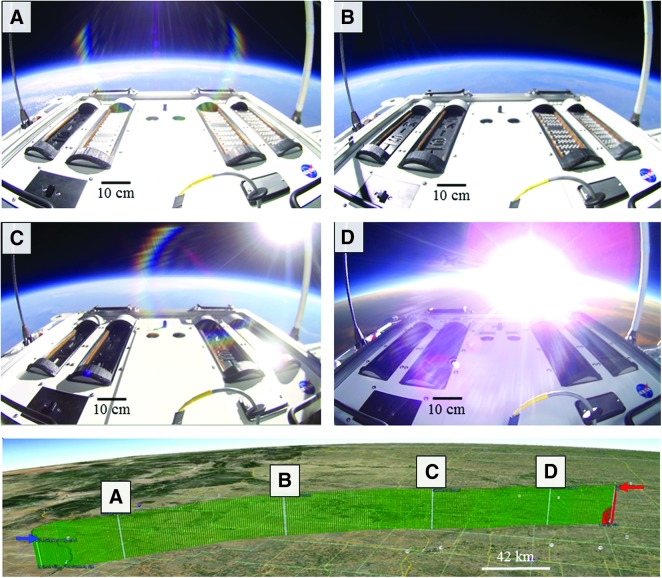
E-MIST flight path on NASA Balloon Program Flight 667NT. Spots visible on nine experimental coupons per skewer base plate were *Bacillus pumilus* SAFR-032 dried spore aliquots. Each sample base plate had one inverted test coupon carrying spores shielded from sunlight. The proxy coupon (visible on the lower right portion of the payload) served as a negative control and also recorded temperature. The upper panel shows the gondola at float 31.4 km ASL and the sequential closure of the payload samples at (**A**) 1819 UTC (Skewer 1); (**B**) 2019 UTC (Skewer 2); (**C**) 2219 UTC (Skewer 3); and (**D**) 0019 UTC (Skewer 4). The lower panel shows ascent, float, and descent trajectory, with skewer closure events labeled across the flight path. The blue arrow marks initial opening of all skewers (at 1619 UTC), and the red arrow marks the start of the gondola descent (11 October 2015 at 0133 UTC). Map credit: “E-MIST Flight Path” 35°20'23.20″N, 102°49'7.15″W. *Google Earth*. 9 April 2013. 8 December 2015.

**FIG. 3.**
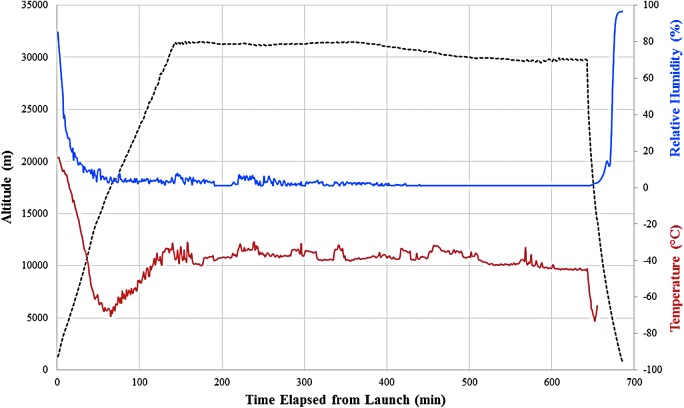
Environmental data for E-MIST flight, showing payload altitude (dashed black) with corresponding atmospheric temperature (red) and relative humidity values (blue). The average atmospheric pressure at the balloon gondola float altitude (which ranged from 29.7 to 31.4 km) was 1.07 kPa.

The E-MIST payload was launched on a high-altitude scientific balloon at 1441 UTC on 10 October 2015 from Fort Sumner, New Mexico (34°29'30″N, 104°13'36″W), traveling 335 km to the northeast for ∼11 h, landing just beyond Amarillo, Texas (36°06'30″N, 100°29'30″W). Samples of *B. pumilus* SAFR-032 spores remained sealed inside the payload until reaching 21.3 km ASL in the lower stratosphere at 1619 UTC, at which point the flight computer rotated the skewers open into the air (Fig. 2). By 1715 UTC, a stable float altitude was achieved, and the payload remained 29.7–31.4 km ASL for a total of 8 h 20 min. The flight computer rotated one skewer to its closed position every 2 h: Skewer 1 closed at 1819 UTC (Fig. 2A), Skewer 2 closed at 2019 UTC (Fig. 2B), Skewer 3 closed at 2219 UTC (Fig. 2C), and Skewer 4 closed at 0019 UTC (Fig. 2D). Environmental data from sensors located inside and outside the E-MIST payload are summarized in Table 1 and Fig. 3. At 0133 UTC (11 October 2015), the gondola was jettisoned from the balloon, returning the payload to the ground on a parachute during a 20 min descent. Personnel from CSBF recovered the payload and transported it back to the launch site facility inside a climate-controlled vehicle. Two days later, flight samples were removed from the E-MIST payload and shipped to the laboratory at KSC (along with unflown ground controls) inside sterile containers at ambient conditions.

**Table 1.** Balloon Flight Environmental Conditions

	*Max.*	*Min.*	*Remarks*
Atmospheric pressure (kPa)	87.6	0.962	Altitude of Ft. Sumner, NM, 1.25 km ASL
Air temp. (°C)	16.2	−73.1	Average air temp. at float was −38.5°C
Payload internal temp. (°C)			Internal heaters pulsed during ascent and descent
Avionics	23.1	−13.3	
Proxy coupon	36.2	−45.9	Average coupon temp. at float was 15.4°C
Battery	14.4	−7.39	
Radiometer	14.1	−6.39	
Battery power (V)	16.7	15.7	
RH (%)	100	<1	Average RH at float altitude was 2.32%
UV (W/m^2^)	N/A	N/A	Data were lost; see text for details
Ground speed (m/s)	33.2	0.010	Average speed at float was 13.6 m/s

**FIG. 4.**
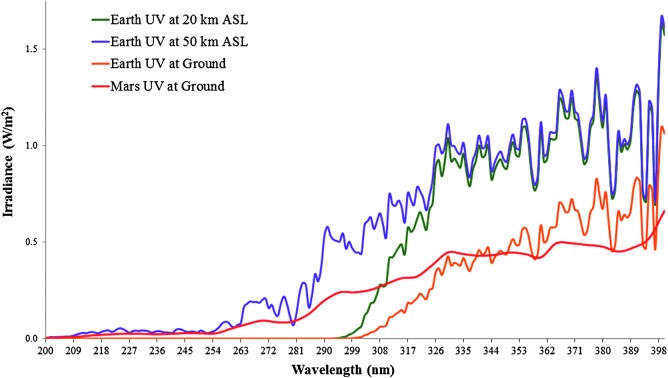
Modeled UV light conditions in Earth's stratosphere (purple and green) closely resemble modeled irradiation levels on the surface of Mars (red) (data from Schuerger *et al.,* 2003), particularly in UVB/UVC wavelengths (100–315 nm). For comparison, modeled ground UV conditions on Earth (at sea level) are depicted in orange, much lower in UVB/UVC due to atmospheric absorbance.

Upon landing, a command to the payload's UV radiometer malfunctioned, preventing the avionics from automatically powering off the instrument. A similar failure occurred during the previous E-MIST test flight (Smith *et al.,* 2014). Briefly, the flight computer received a false indication that the radiometer was shut down; however, the instrument continued to record data until the payload was manually powered down 6 days later at KSC. The radiometer instrument could only store 72 h of measurements. Consequently, the flight UV data were overwritten and unrecoverable. Since a subsequent experiment could not be flown, we remained reliant upon modeling from previously acquired UV measurements in the stratosphere to understand the likely dose of irradiation. To provide a range of expected UV values for the 21.3–31.4 km ASL mission float profile, we constructed a model framed between 20 and 50 km ASL altitudes, based on previous calculations (Smith *et al.,* 2011). Model inputs used the E-MIST balloon flight path latitude (34–36°N) and UV values measured by the Nimbus-7 Solar Backscatter UV instrument that took solar spectra while ascending through the stratosphere in January 1979 (McPeters *et al.,* 1984, 1996). Ultraviolet attenuation was determined by using previously established ozone concentration values acquired in the 30–40°N latitude range by monthly satellite observations (McPeters *et al.,* 2007). Ozone thickness measurements averaged out to 303.1 DU, 214.1 DU, and 0.858 DU for ground, 20 km ASL, and 50 km ASL, respectively, and were converted to absorption coefficient factors for the model. Dosages were calculated to provide a total, instantaneous flux rate (in W/m^2^) for UVA (315–400 nm), UVB (280–315 nm), and UVC (100–280 nm) with two simplifying assumptions: (1) direct irradiation (*i.e.,* no scattering) and (2) a fixed solar zenith angle of 30°. For the 20 km ASL estimation, we used a temperature of −45°C with atmospheric pressure at 5.57 kPa; for the 50 km ASL estimation, we used a temperature of 0°C with atmospheric pressure at 0.101 kPa. Modeled quantities are summarized in Table 2 and Fig. 4.

**Table 2.** Estimated UV Rates (Instantaneous)

	*UVA total (W/m*^2^*)*	*UVB total (W/m*^2^*)*	*UVC total (W/m*^2^*)*	*Total (W/m*^2^*)*
	*315–400 nm*	*280–315 nm*	*100–280 nm*	*100–400 nm*
Ground (Earth)	44.1	1.01	0	45.1
20 km ASL (Earth)^a^	82.4	4.16	0.00550	86.6
50 km ASL (Earth)	86.2	17.1	5.20	109
Ground (Mars)^b^	39.0	8.38	3.18	50.6

^a^ Modeled stratosphere UV data from Smith *et al.* (2011).

^b^ Modeled Mars ground UV data from Schuerger *et al.* (2003).

### 3.2. Survival of *Bacillus pumilus* SAFR-032

**FIG. 5.**
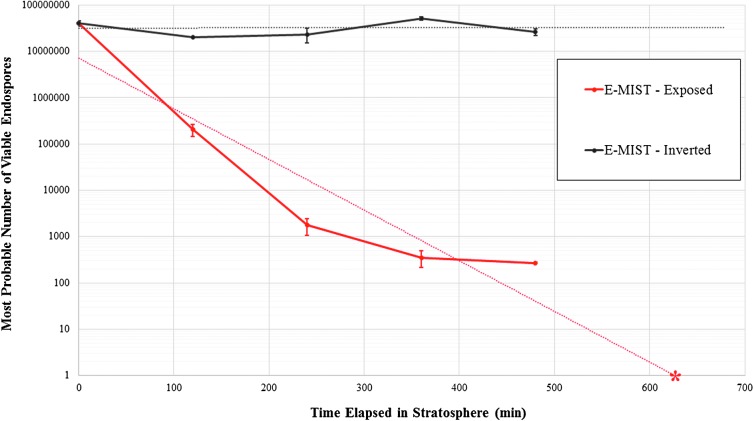
Inactivation rate of *B. pumilus* SAFR-032 spores in Earth's stratosphere over the 8 h experiment. Note: average MPN values shown on a logarithmic scale. Experimental control coupons contained approximately 4.07 × 10^7^ spores per aliquot. Black values (*N* = 3 per time group) represent the inverted flight coupons exposed to stratospheric conditions without sunlight; red values (*N* = 8–9 per time step) show the exposed flight coupons (*P* < 0.0001). Dashed trend lines were added to the data series, depicting the relative stability of inverted samples and the estimated 630 min required for total inactivation of exposed samples (red asterisk).

Coupons that were flown to the stratosphere but inverted (shielding bacterial spores from sunlight) did not change significantly over the course of the 8 h experiment (Fig. 5). MPN estimates for inverted flight coupons ranged from 2.00 × 10^7^ to 5.17 × 10^7^ spores per aliquot, which were values similar to transport and ground control coupons (4.02 × 10^7^ and 4.12 × 10^7^ viable spores per aliquot, respectively). In contrast, spores flown to the stratosphere and exposed to sunlight changed significantly across the experiment compared to controls (*F* = 11.52, *P* < 0.0001). Coupons exposed for 2 h (Skewer 1) dropped by 2 orders of magnitude to 2.04 × 10^5^ viable spores (*N* = 9). Rapid inactivation continued, with viable spores declining another 2 orders of magnitude to 1.76 × 10^3^ (*N* = 9) by the 4 h time step (Skewer 2). The difference between Skewer 1 and 2 samples was significant (*P* < 0.01). Another 2 h in the stratosphere resulted in a more gradual, though still significant (*P* < 0.01), inactivation rate with viability values at the 6 h exposure group (Skewer 3) dropping an additional order of magnitude to 353 spores (*N* = 9). Only 267 viable spores (*N* = 8), or 0.0007% of the *B. pumilus* SAFR-032 spore quantity initially seeded onto coupons, were recovered from the final sample set exposed to the stratosphere for 8 h (Skewer 4). One outlier from the 8 h group (MPN value of 8.60 × 10^3^) was discarded due to a suspected MPN processing error. The overall decline (5 orders of magnitude) between control samples and Skewer 4 was strongly significant (*P* < 0.01); however, the smaller difference between Skewer 3 and 4 groups (86 viable spores, on average) was not significant based on a Tukey HSD test. Notably, none of the sample coupons exposed to the stratosphere for 8 h were completely sterilized. In fact, the lowest viability estimate from a single aliquot processed was 200 spores. To forecast the amount of time in the stratosphere needed for complete inactivation, a trend line was calculated by using the survivability decay rate from exposed flight coupons. Based on this projection, no viable spores would remain if flight samples had an additional 150 min of Sun exposure in the stratosphere (630 min total time).

When compared to the Sun-exposed stratosphere coupons, the unchanged survival of spores harvested from the inverted stratosphere coupons revealed that UV irradiation was responsible for bacterial inactivation (*i.e.,* not extreme cold, dryness, or hypobaria). Thus, we conducted a series of ground-based UV studies in the laboratory to determine how the initial starting concentration of bacterial spores might influence survivability. Our tests varied the intensity and duration of UVC illumination by changing exposure time, distance from the light source, and incidence angle to coupon surfaces. Samples that were identical to flight coupons (prepared at the same time with a concentration of 4.52 × 10^7^ spores) did not change significantly (*F* = 3.69, *P* > 0.01) when illuminated with 0.27–0.5 W/m^2^ of 271.1 nm UVC for up to 4 h. MPN estimates (*N* = 9 with each treatment) for exposure times of 1.3, 6.7, 50, 100, and 240 min were stable and generally within standard error ranges at 2.82 × 10^7^, 3.96 × 10^7^, 6.96 × 10^7^, 1.78 × 10^7^, and 1.13 × 10^7^ spores, respectively.

**FIG. 6.**
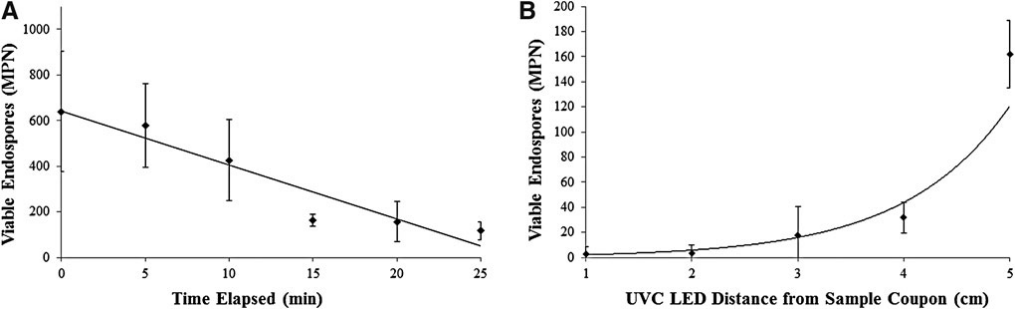
(**A**) Survivability of *B. pumilus* SAFR-032 spores exposed to 0.03 W/m^2^ of 271.1 nm UVC for 0–25 min at 5 cm and 90° angle of incidence (*R*^2^ = 0.911; *N* = 3). (**B**) Survivability of *B. pumilus* SAFR-032 spores exposed to 0.03–0.5 W/m^2^ of 271.1 nm UVC for 15 min at 90° angle of incidence and distances 1–5 cm from LED source (*R*^2^ = 0.950; *N* = 3).

A different pattern was observed for our low-concentration ground tests when using only ∼640 *B. pumilus* SAFR-032 spores per coupon (*N* = 3) and exposing samples to 0.03 W/m^2^ of 271.1 nm UVC. We measured a significant survivability reduction by 15 min (*F* = 5.41, *P* = 0.014) and an overall negative correlation (*R*^2^ = 0.911) between UVC exposure time and spore viability (Fig. 6A). Not all spores were inactivated at the longest exposure period of 25 min; approximately 117 remained. When the incidence angle of UVC illumination (relative to the sample coupons) was tested at 90°, 75°, 60°, and 45° for experiments in which 0.03 W/m^2^ of UVC were used, no significant survivability changes between groups were observed (data not included)—all groups declined at a similar rate. In a final 15 min experiment in which the same starting concentration of spores and a 90° incidence angle were used, bacterial inactivation was greater when the UVC light source was closer to the sample coupon (*R*^2^ = 0.950) (Fig. 6B). The decline across groups was significant (*F* = 15.4, *P* = 0.00028), and viable spores were only recovered from 33% of sample coupons located 1 cm away from the LED.

Finally, we performed a scanning electron microscope analysis to better understand the distribution and arrangement of bacterial spores deposited on aluminum test coupons at a concentration of ∼4.07 × 10^7^ spores per 20 μL aliquot (see Fig. 1B). Random areas of the dried bacterial aliquots were surveyed, and the micrographs revealed complete sporulation (*i.e.,* no vegetative *B. pumilus* SAFR-032 cells) with a predominantly monolayered arrangement of clustered spores on the coupon surface. Where stacking occurred, it generally appeared about 2–3 spores thick. No layering was noticeable for the ground test coupons prepared at a lower concentration (∼640 spores per aliquot). Since MPN enumeration depended upon the full removal of spores from experimental coupons, a subset of test coupons was examined after PVA peel processing to determine whether any spores were left behind; none were observed. In addition, PVA chemistry did not affect the viability of spores—a control test resulted in the same MPN range for identical spore aliquots with and without PVA peels.

### 3.3. Resequencing of *Bacillus pumilus* SAFR-032

Using germinated spores that survived the stratosphere (from exposed and inverted sample groups), we performed a nucleotide variant analysis focusing on known coding regions within the *B. pumilus* SAFR-032 genome. After resequencing the samples, we used the CLC Workbench to identify deletions, insertions, and substitutions compared to the reference genome. The mapping analysis was also done for unflown control coupons. Only *B. pumilus* SAFR-032 sequences were detected during the sequencing analysis, indicating that microbial contamination was not an issue in the laboratory or field. Resequencing produced a total of 87 nucleotide variants at sample frequencies ranging from 33% to 100%, most of which were from the same coding region: deletions at ABV62232.1 (A/-), ABV62537.1 (T/-), ABV62702.1 (T/-), ABV62978.2 (G/-), ABV63654.1 (A/-); insertions at ABV62978.2 (-/G), ABV63866.1 (-/T); and substitutions at ABV61973.1 (T/G), ABV62475.1 (G/T), ABV62978.2 (C/A), ABV63728.1 (T/A), ABV63728.1 (G/A), ABV63728.1 (C/A), ABV60913.1 (C/T), and ABV61863.1 (C/A). Since these variants appeared with both flight and ground control sequences, we removed them from our analysis in order to focus strictly on unique changes for flight samples. The nucleotide variants—all single base pair substitutions—were observed in the 2 h exposed group [ABV61341.1 (A/T) at 23.8% frequency] and the inverted flight coupon group [ABV63490.1 (C/T) at 39.3% frequency; ABV63868.1 (C/T) at 27.6% frequency]. Upon publication, raw sequencing results will be archived in the NASA GeneLab repository (http://genelab.nasa.gov) for the scientific community to access.

## 4. Discussion

We conducted a mixture of flight and ground experiments examining bacterial spore survival within a Mars analog environment high above Earth's surface. After enduring intense selective pressures used in spacecraft assembly facilities and with natural polyextremophilic resistance, *B. pumilus* SAFR-032 spores could be pre-adapted to the harsh conditions of spaceflight and capable of reaching Mars unharmed (Link *et al.,* 2004; Ghosal *et al.,* 2005; Kempf *et al.,* 2005; Gioia *et al.,* 2007; Vaishampayan *et al.,* 2012; Tirumalai *et al.,* 2013). Besides the *B. pumilus* SAFR-032 isolate, numerous planetary protection studies have documented the diversity of microorganisms on spacecraft surfaces and in clean rooms (Link *et al.,* 2004; La Duc *et al.,* 2009, 2012; Vaishampayan *et al.,* 2013; Benardini *et al.,* 2014). However, without samples returned from Mars, it is not possible to measure the survival of unintentionally introduced terrestrial contamination. Instead, specimens sent to Earth's stratosphere can be analyzed after exposure to a similar environment. Our balloon study challenged *B. pumilus* SAFR-032 spores to conditions reaching about 1 kPa, −73°C, <1% RH, and UV levels totaling 86.6–109 W/m^2^ for up to 8 h.

### 4.1. Flight and ground experiment implications

Flight results reveal a rapid inactivation of *B. pumilus* SAFR-032 spores in the stratosphere. Viability was reduced by >99.9% after only 8 h of exposure to the stratospheric conditions with sunlight. Spores subjected to identical conditions without sunlight (*i.e.,* inverted flight coupons) did not decline significantly. Thus, we can conclude that solar radiation was the leading factor influencing *B. pumilus* SAFR-032 spore survival in the stratosphere. Reduced atmospheric pressure, temperature, and water availability were not biocidal to the spores. Our results are consistent with other Mars simulation survivability investigations (Schuerger *et al.,* 2003, 2006; Cockell *et al.,* 2005; Diaz and Schulze-Makuch, 2006; Tauscher *et al.,* 2006; de la Vega *et al.,* 2007; Moores *et al.,* 2007; Osman *et al.,* 2008; Fendrihan *et al.,* 2009; Smith *et al.,* 2009; Gómez *et al.,* 2010; Peeters *et al.,* 2010; Johnson *et al.,* 2011; Kerney and Schuerger, 2011). For instance, Schuerger *et al.* (2003) showed that monolayered bacterial spores exposed to simulated martian conditions in an environmental chamber were inactivated after 3 h. Collectively, our flight results and other experiments predict a low probability of bacterial persistence on the surface of Mars—provided that contaminating microbes are directly illuminated by sunlight. Even though the solar constant at Mars is only ∼43% that at Earth, the surface of the Red Planet under clear-sky conditions has roughly 3 orders of magnitude higher UV irradiance than the surface of Earth (Cockell *et al.,* 2000; Nicholson *et al.,* 2005), particularly in biocidal UVC wavelengths (100–280 nm). This is due to the rarified martian atmosphere (∼0.7 kPa), with fewer chemical species capable of UV attenuation (Zurek *et al.,* 1992); about 95% of the martian atmosphere is CO_2_, which leads to solar radiation absorbance more efficiently at wavelengths <190 nm (Kuhn and Atreya, 1979). Nicholson *et al.* (2005) discussed the resistance and susceptibility of bacteria to UVC and described it as ≥300-fold more effective at damaging DNA and killing bacterial spores than UVB and UVA.

Since short UV wavelengths predominantly determine the fate of microbes delivered to Mars, artificial solar radiation light sources used in past survival simulation studies (Schuerger *et al.,* 2003, 2006; Cockell *et al.,* 2005; Diaz and Schulze-Makuch, 2006; Tauscher *et al.,* 2006; de la Vega *et al.,* 2007; Moores *et al.,* 2007; Osman *et al.,* 2008; Fendrihan *et al.,* 2009; Smith *et al.,* 2009; Gómez *et al.,* 2010; Peeters *et al.,* 2010; Johnson *et al.,* 2011; Kerney and Schuerger, 2011) must be carefully considered. No single lamp or group of lights can fully simulate the spectrum of wavelengths expected at the martian surface. It is also difficult to mimic a drifting solar zenith and hardware surface scattering effects inside the restrictive confines of an environmental chamber. Thus, most ground survival simulation studies bathe microorganisms continuously to radiation at a fixed angle of incidence within a narrow band of UV. Light does not behave this way in nature. Earth's stratosphere can be used to provide a more dynamic illumination with UV doses roughly equivalent to levels expected on the martian surface, as an alternative approach to the inherent limitations associated with laboratory studies. At its mean orbital distance, Mars is thought to have fluence rates on the surface around 3.18 and 8.38 W/m^2^ for UVC and UVB, respectively (Nicholson *et al.,* 2005). In comparison, the stratosphere UV model developed for this study generated similar levels for UVC and UVB at 20–50 km ASL: 0.00550–5.20 and 4.16–17.1 W/m^2^, respectively. Our model was consistent within the range of measured values by Kylling *et al.* (2003), who flew a UV radiometer to 30.5 km ASL in the stratosphere over France (∼44.5°N), recording measurements at 312 and 340 nm across a solar zenith range of 76–94° in flight.

### 4.2. Significance of bioburden configuration and concentration

Since Mars-bound, robotic spacecraft missions are required to reduce bioburden, lingering contaminants would likely be in low concentrations across hardware components. For instance, the total bioburden on exposed surfaces of the landed Mars Science Laboratory hardware was estimated at 5.64 × 10^4^ spores (22 spores/m^2^), with only 1.57 × 10^4^ spores estimated on the rover itself (Benardini *et al.,* 2014). Comparatively, our dried 20 μL aliquots contain approximately 41 million spores, which is several orders of magnitude higher than typical bioburden for cleaned spacecraft surfaces. While our concentration could be considered a “nightmare” contamination scenario, we still measured near-complete bacterial inactivation (>99.9%) after 8 h in the stratosphere using one of the most UV-resistant bacterial strains recovered from a spacecraft clean room to date (Link *et al.,* 2004; Gioia *et al.,* 2007; Vaishampayan *et al.,* 2012; Tirumalai *et al.,* 2013). With our experimentally derived kill curve, we forecasted a complete spore inactivation in the stratosphere with only 630 min of Sun exposure.

For our results to be useful for predicting the response of landed Mars bioburden on spacecraft, a few key assumptions would be required: (1) that stratospheric conditions resemble the surface environment on Mars; (2) that a homogeneous monolayer of spores with physiology similar to *B. pumilus* SAFR-032 would be distributed across spacecraft external surfaces; and (3) that no dust or hardware components shade bacterial contaminants. It is already known that the biocidal effects from radiation are mostly applicable to surface or shallow subsurface contamination (Nicholson *et al.,* 2005). Schuerger *et al.* (2003) and Cockell *et al.* (2005) found that the survival of bacteria increased significantly when shielded from UV irradiation by thin layers of dust or rocks. In fact, *Chroococcidiopsis* sp. 029 retained viability after 8 h under rock coverage only 1 mm thick (Cockell *et al.,* 2005), and *B. subtilis* survived 8 h of UV irradiation when covered by only a 0.5 mm coating of dust (Schuerger *et al.,* 2003). Similarly, when *B. pumilus* SAFR-032 spores were combined with 60 μm Mars regolith soils and incubated in a simulated martian atmosphere for 24 h, only a 4-log reduction in viability was reported (Osman *et al.,* 2008). Overlying dead biomass can provide another means of UV shielding to microorganisms in layers below. Orbital experiments outside the ISS concluded *B. pumilus* SAFR-032 spores were capable of tolerating Mars-like radiation dosages combined with the vacuum of space for 18 months probably due to such shielding (Horneck *et al.,* 2012; Moeller *et al.,* 2012; Nicholson *et al.,* 2012; Vaishampayan *et al.,* 2012). Desiccated layers 2–3 spores thick of *B. pumilus* SAFR-032 were used for the ISS study (Horneck *et al.,* 2012), and we used the same order of magnitude with our balloon mission to the stratosphere. Predictably, the two experiments had similar outcomes for Sun-exposed spores. Outside the ISS, the *B. pumilus* SAFR-032 survival rate was less than 10^−6^ (Horneck *et al.,* 2012). A small, though noteworthy, number of spores persisted in both experiments conducted in the stratosphere and outside the ISS. Either a subset of bacteria was more resistant to biocidal conditions, or more likely, lethal wavelengths of sunlight were attenuated by dead spore layers, clustering, or microscopic pits and cracks in the aluminum coupon surface (Schuerger *et al.,* 2005; Horneck *et al.,* 2012).

To further examine the response of individual spores to irradiation, we performed a standalone UVC experiment with a lower concentration of spores to eliminate layering and clustering. Our starting concentration was the same order of magnitude as balloon flight survivors. The ground experiment showed rapid inactivation within 25 min, even at UVC levels (0.03–0.5 W/m^2^) lower than what spores would experience in the stratosphere, outside the ISS, or on the surface of Mars. When the same ground experiment was performed for up to 4 h using a higher concentration (4.52 × 10^7^ spores per aliquot), the UVC had no significant effects on viability compared to controls. From these supplementary ground studies, we concluded that the concentration and layering configuration of bacterial spores on sample surfaces primarily determined survivability. It is also worth emphasizing how efficiently UVC LEDs sterilized small amounts of bacterial bioburden in our ground experiment. This relatively new technology could be a low-mass, low-power solution to the problem of spores buried deep within spacecraft hardware, otherwise inaccessible to cleaning efforts and fully shielded from sunlight at Mars. Our single UVC LED produced biocidal action (>95%) within 15 min at distances ranging 1–5 cm from illuminated surfaces. Vehicles someday sent to Mars special regions (Rettberg *et al.,* 2016) could be designed with larger, more sophisticated UVC LED arrays embedded in spacecraft hardware most likely to touch the martian regolith (*e.g.,* drill bits and wheels).

### 4.3. Genomic consequences of Mars-like conditions on *B. pumilus* SAFR-032

Spores surviving our flight experiment could have been a stochastic result or beneficiaries of coupon cracks and overlying dead biomass. But another possibility worth examining was whether a genomic advantage enabled persistence. To test this hypothesis, we resequenced surviving *B. pumilus* SAFR-032 spores to look for nonlethal mutations in coding regions of the genome. Common nucleotide variants from flight and ground samples were identified. After subtracting these variants from the analysis, three single nucleotide polymorphisms remained from the 2 h exposed population at ABV61341.1 (A/T) and the inverted flight coupon population at ABV63490.1 (C/T) and ABV63868.1. The first region, ABV61341.1, is thought to be associated with producing ABC transporter proteins (Gioia *et al.,* 2007). ABV63490.1 is a well-conserved region within Firmicutes and possibly involved with the initiation of sporulation and resistance of bacteria to extreme pH, temperature, and hypersaline conditions. Similarly, ABV63868.1 codes for an amidase protein that may guide sporulation and other metabolic pathways (Gioia *et al.,* 2007; Krulwich *et al.,* 2009). The total number of single nucleotide polymorphisms detected was fairly small considering the genome size surveyed (3.7 Mbp). However, lethal changes to the genome should not have been detected by our assay, which targeted sequences from the surviving subset of spores. Moreover, *Bacillus* sp. have cellular systems for repairing UV damage to DNA. Spore photoproduct lyase or recombinatorial and nucleotide excision repairs in germinating spores (reviewed by Nicholson *et al.,* 2005) might explain the relatively few nucleotide variants identified through resequencing. While variants were relatively uncommon in our samples, base pair substitutions in three coding regions associated with bacterial sporulation and metabolism do warrant further investigation. Selective pressures in Earth's upper atmosphere, in transit to Mars, or on the surface of the Red Planet could feasibly establish nonlethal mutations that alter bacterial gene pools deposited in new environments. Functional gene changes that result in heightened *B. pumilus* SAFR-032 resilience were not tested in this study, but comparative transcriptomics, proteomics, and metabolomics are targets of future investigation exploring the molecular basis of polyextremophiles. Intriguingly, previous research has shown that UVC resistance doubled in *B. pumilus* SAFR-032 spores exposed to the ISS space environment for 18 months (Vaishampayan *et al.,* 2012). Induced mutations to rifampicin resistance and sporulation deficiency were also observed to increase by several orders of magnitude for spores exposed outside the ISS (Moeller *et al.,* 2012).

### 4.4. Earth's stratosphere as a Mars analog environment

While our study focused on the forward contamination of Mars, Earth's stratosphere also provides opportunities for multicellular biological investigations focusing on the destructive effects of ionizing radiation (Cucinotta *et al.,* 2001; Schimmerling, 2007). In fact, 2 weeks before the E-MIST launch, a suite of ionizing radiation instruments was sent to 36.7 km ASL in the stratosphere from Ft. Sumner, New Mexico (Flight #666N). The Radiation Dosimetry Experiment (RaD-X) measured ionizing dose rates of approximately 0.066 mGy/day with a Liulin LET spectrometer (Mertens *et al.,* 2016). For comparison, recent measurements by the Mars Science Laboratory rover detected an ionizing radiation flux of approximately 0.18–0.225 mGy/day on the surface of Mars (Hassler *et al.,* 2014). Antarctic balloon flights would provide even greater levels of ionizing radiation due to the window of energy-intense particles that penetrate stratospheric latitudes above 70° (Adams *et al.,* 2007). Moreover, circumpolar flights now offer long-duration NASA balloons capable of staying aloft for 100 days (Jones, 2014). Advantages of using the stratosphere as a Mars analog environment instead of ground-based hypobaric chambers, particle accelerator facilities, or orbital experiments in space can be summarized to include (1) seasonal balloon flight opportunities from multiple locations around the world, (2) logistical simplicity with biological experiments (late loading and a rapid return of samples to the laboratory), (3) relative affordability and rapid development compared to other flight-based investigations, and most importantly (4) a realistic, dynamic radiation spectrum naturally paired with other Mars-like conditions (extreme cold, dryness, and hypobaria).

### 4.5. Limitations and future directions

An unexpected loss of radiometer data from our test flight made a direct analysis between UV levels in the stratosphere and *B. pumilus* SAFR-032 spore survival unachievable. This includes possible effects of transient shadows cast by the balloon flight train (as the gondola rotated in the stratosphere). Also, total illumination on sample coupons likely decreased as the Sun set on the horizon toward the end of the experiment (see Fig. 2D). The inactivation rate of the spores decelerated with the final two time steps (6 h, Skewer 3; 8 h, Skewer 4), probably due to the setting Sun, but without UV radiometer measurements such direct correlations could not be established in our study. Future missions will aim to fly on a balloon gondola flown in the polar stratosphere at summertime to enable continuous, uninterrupted sunlight and longer-duration exposures. To prevent another UV radiometer command malfunction, backup data will be stored off-instrument. The next-generation E-MIST payload system will also aim to incorporate a dosimeter for measuring ionizing radiation levels in the stratosphere. Until a reflight opportunity for the E-MIST payload, independent radiation measurements from the stratosphere can be used for evaluating the fidelity of the UV model developed herein (*e.g.,* Kylling *et al.,* 2003; Mertens *et al.,* 2016). Our forecast suggested that stratosphere UVB/UVC levels (averaged between 20 and 50 km ASL) would be similar to martian conditions calculated by Schuerger *et al*. (2003)—approximately 10 and 3 W/m^2^ for UVB and UVC, respectively, from each modeled environment. This agreement for fluence rates between environments is important because short-wavelength UV is the most effective biocidal wavelength for spore-forming bacteria (Nicholson *et al.,* 2005). While modeled rates from 315 to 400 nm were higher for the stratosphere (∼84 W/m^2^) than expected on Mars (∼39 W/m^2^), UVA would not have the same influence on bacterial survival.

Our experiment tested only one spacecraft assembly facility isolate, *B. pumilus* SAFR-032, due to its unique resistance to environmental extremes, including radiation, and its use in similarly scoped studies (Horneck *et al.,* 2012; Moeller *et al.,* 2012; Nicholson *et al.,* 2012; Vaishampayan *et al.,* 2012). Subsequent flights evaluating other clean-room-archived isolates would be useful to determine which contaminants might be problematic if viably landed on Mars. Since the *B. pumilus* SAFR-032 response to the stratosphere environment was unknown prior to our balloon flight, we prepared samples at concentrations much higher than what would be reasonably expected for a Mars spacecraft prepared in a clean room. Our results reveal that spore stacking could have prolonged survival for a subset of the stratosphere-flown population. Another limitation of our experimental design was that spores were deposited on a relatively flat metal surface for Sun exposure. Actual spacecraft components where terrestrial contaminants could linger would have a more complex configuration, and the effects of martian dust buffering sunlight are unknown. Consequently, the need for evaluating more complicated, though realistic, bioburden distributions with future E-MIST experiments is a high priority for our team.

## 5. Conclusion

We observed a >99.9% inactivation for Sun-illuminated bacterial spores exposed to Mars-like conditions in the stratosphere for 8 h. Our starting concentration of viable spores was substantially higher than contamination levels typical for Mars-bound spacecraft, and we used one of the most radiation-resistant bacterial strains known to be in clean rooms; nevertheless, *B. pumilus* SAFR-032 spores were rapidly killed by sunlight in the stratosphere. Survivors were most likely lingering below layers of overlying dead spores or within small surface defects on the experimental coupons. Our flight results and supplementary ground experiments suggest that the concentration of spores and their distribution on spacecraft hardware primarily determined survival rates. Based on our experimental observations, it seems unlikely that a fully exposed, sunlit bioburden sent to Mars could perpetually withstand the effects of UV radiation at the Red Planet's surface. Using this balloon study in the stratosphere as a stand-in for the martian environment, we predict that most Sun-exposed bacterial contaminants at a level below ∼10,000 spores would be inactivated within 1 sol. Future balloon-based missions in the stratosphere can be used to continue studying other spacecraft clean-room isolates, with and without dust coverage, and at lower concentrations more representative of spacecraft bioburden. Outcomes from such experiments could be used to develop species-specific inactivation models and may reveal genes or cellular mechanisms bestowing exceptional microbial resistance to environmental extremes, including conditions expected on Mars.

## Supplementary Material

Supplemental data

Supplemental data

Supplemental data

## Abbreviations Used

ASL: above sea level
CSBF: Columbia Scientific Balloon Facility
E-MIST: Exposing Microorganisms in the Stratosphere
ISS: International Space Station
KSC: Kennedy Space Center
LED: light-emitting diode
MPN: most probable number
PVA: polyvinyl alcohol
