# Supplementary material for: Stratosphere Conditions Inactivate Bacterial Endospores from a Mars Spacecraft Assembly Facility
Source: Astrobiology. 2017 Apr 1;17(4):337–50. doi: 10.1089/ast.2016.1549 (PMC5399745; doi:10.1089/ast.2016.1549)
Supplement: Supplemental data [file Supp_Figure1.pdf]

Supplementary Figures

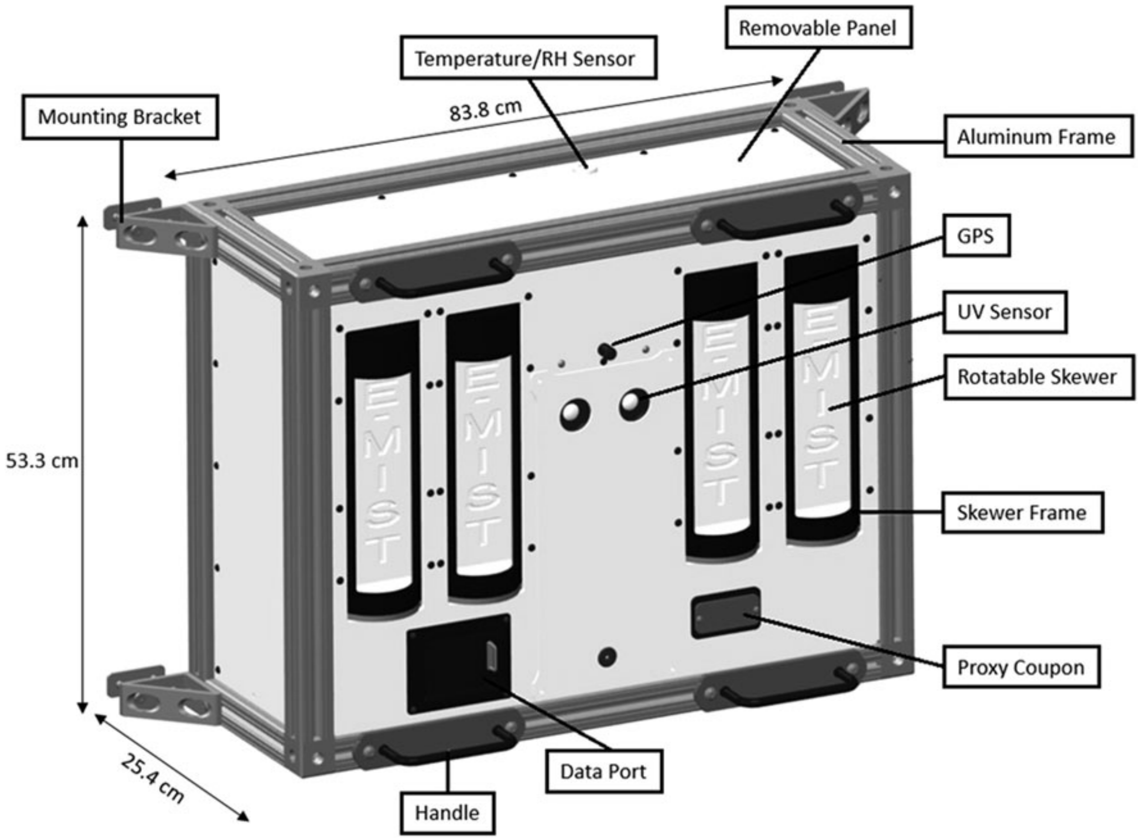

FIG. S1. Labeled isometric model of the re-designed Exposing Microorganisms in the Stratosphere (E-MIST) payload with a mass of 36 kg. The system mounts to NASA scientific balloon gondolas and has 4 independently rotating sample canisters (''skewers'') controlled by a flight computer.
