# Supplementary material for: Stratosphere Conditions Inactivate Bacterial Endospores from a Mars Spacecraft Assembly Facility
Source: Astrobiology. 2017 Apr 1;17(4):337–50. doi: 10.1089/ast.2016.1549 (PMC5399745; doi:10.1089/ast.2016.1549)
Supplement: Supplemental data [file Supp_Figure2.pdf]

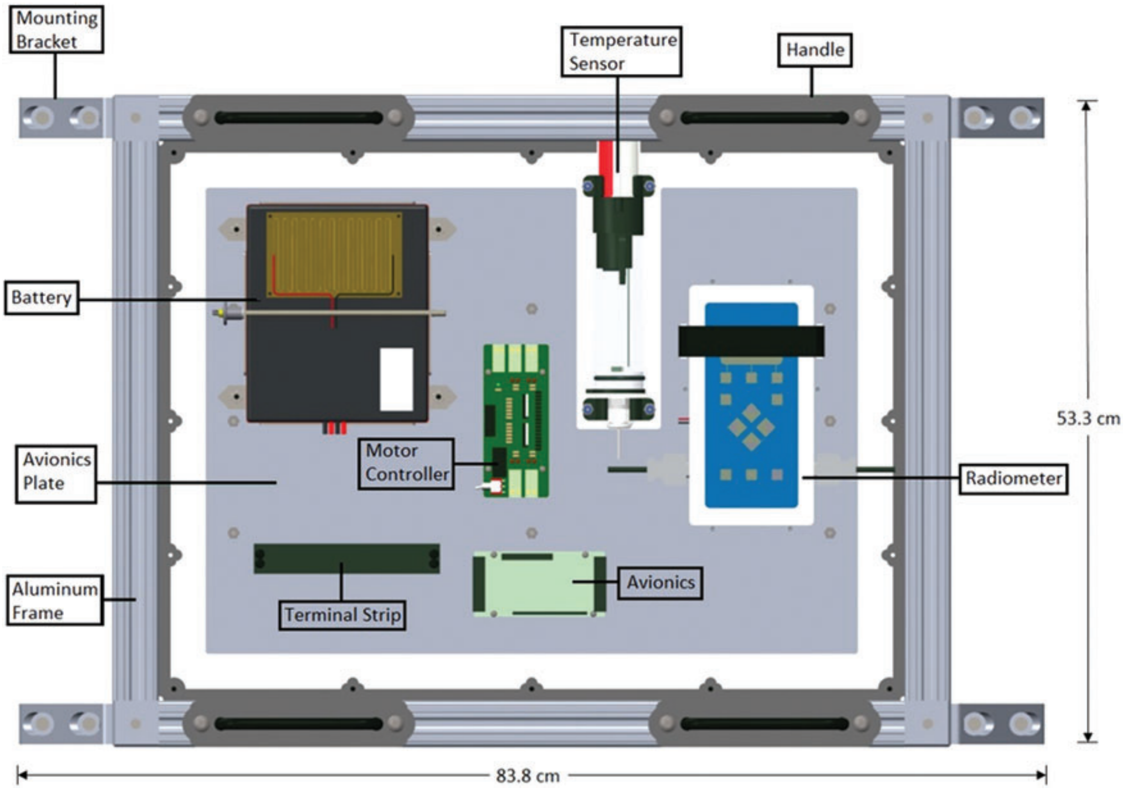

FIG. S2. Labeled model of internal components for the re-designed E-MIST payload, showing power supply, instrument, and computer configuration.
