# Supplementary material for: Stratosphere Conditions Inactivate Bacterial Endospores from a Mars Spacecraft Assembly Facility
Source: Astrobiology. 2017 Apr 1;17(4):337–50. doi: 10.1089/ast.2016.1549 (PMC5399745; doi:10.1089/ast.2016.1549)
Supplement: Supplemental data [file Supp_Figure3.pdf]

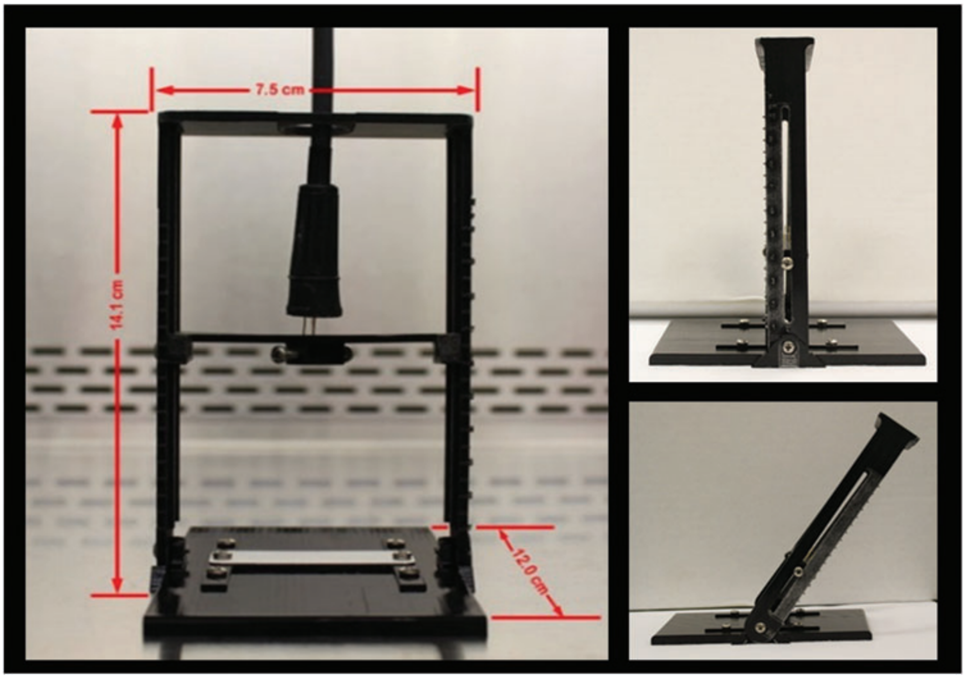

FIG. S3. Custom-built test stand for performing ground UVC irradiation studies. Experimental coupons with quantities of *B. pumilus* SAFR-032 spores were mounted on the base plate below the test stand bridge. The distance and angle of the UVC LED could be modulated as depicted in the right panel.
